# Elephant Rewilding Indirectly Affects Rodent Site‐Use and Behavior by Moderating Coarse Woody Debris Density

**DOI:** 10.1002/ece3.73928

**Published:** 2026-08-03

**Authors:** Christopher E. Gordon, Paul Allin, Michelle Greve, Jens‐Christian Svenning

**Affiliations:** ^1^ Hawkesbury Institute for the Environment Western Sydney University Penrith New South Wales Australia; ^2^ Center for Ecological Dynamics in a Novel Biosphere (ECONOVO), Department of Biology Aarhus University Aarhus C Denmark; ^3^ Transfrontier Africa Hoedspruit South Africa; ^4^ Department of Plant and Soil Sciences University of Pretoria Pretoria South Africa

**Keywords:** megafauna, reintroduction time, rodent, savannah, South Africa, trophic rewilding

## Abstract

Megaherbivores such as African savannah elephants ( *Loxodonta africana* ) are key ecosystem engineers that modify vegetation structure through processes including tree felling and branch breakage. However, the cascading effects of these structural changes on smaller fauna remain poorly understood. Here, we examined how elephant rewilding influences coarse woody debris (CWD) density and, in turn, the behavior of a common rodent, the Natal multimammate mouse ( *Mastomys natalensis* ), in lowveld savannah, South Africa. Using field surveys and manipulative experiments, we quantified (i) CWD abundance across reserves where elephants had and had not been restored (henceforth “rewilded”) by 2003, and (ii) rodent site visitation, behavior, and giving‐up density (GUD) within CWD and adjacent open microhabitats. Bayesian models showed that medium‐ and large‐sized CWD density was on average 5.5 and 18.0 times higher where elephants had been restored than not. Rodent visitation, feeding, and resting behaviors were on average 1.66, 1.76, and 7.75 times higher in CWD than open areas, while GUD values were on average 1.1 times higher in open than CWD areas, indicating reduced perceived predation risk in CWD. These findings suggest that elephant‐induced habitat modifications create structural refugia that influence rodent foraging and risk perception, consistent with the “landscape of fear” framework. By altering fine‐scale habitat heterogeneity, elephants indirectly influence small‐mammal behavior, which is likely to affect processes such as seed predation and secondary dispersal. Our results provide evidence that megafauna rewilding can reinstate lost trophic interactions and behaviorally mediated ecosystem functions, emphasizing the importance of considering cross‐trophic behavioral pathways when evaluating restoration outcomes.

## Graphical abstract

Megaherbivores such as African savannah elephants are ecosystem engineers that modify vegetation structure, yet the cascading effects of these changes on smaller fauna remain poorly understood. We examined how elephant rewilding affected coarse woody debris (CWD) density and the behavior of the Natal multimammate mouse in lowveld savannah, South Africa, using field surveys and manipulative experiments to quantify CWD abundance, rodent visitation and behavior, and giving‐up density (GUD) across rewilded and non‐rewilded reserves. Elephant restoration increased medium‐ and large‐sized CWD density (5.5‐ and 18‐fold, respectively), and rodents showed higher visitation, feeding, and resting in CWD with lower GUDs relative to open areas, indicating that elephant‐driven habitat modification creates structural refugia benefiting smaller fauna.

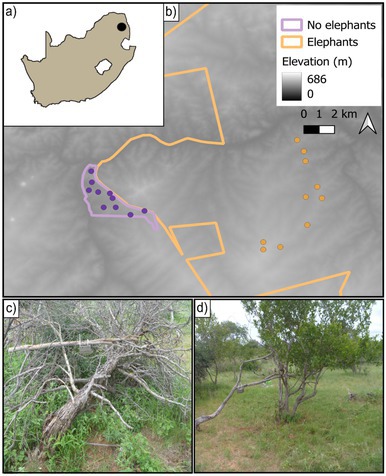

## 1 Introduction

Trophic rewilding is a conservation approach that promotes the reintroduction or recovery of historically depauperate faunal communities to restore ecosystem function and support resilient, self‐regulating biodiverse ecosystems (Svenning et al. 2016, 2024). Central to this concept is the reinstatement of species or faunal assemblages that closely resemble evolutionary baselines, with the aim of re‐establishing lost ecological interactions and processes. Trophic rewilding has largely focused on large‐bodied megafauna because of their disproportionate influence on ecosystems through trophic regulation and ecosystem engineering processes (Bakker et al. 2016; Svenning et al. 2016). Although experimental and observational studies investigating rewilding outcomes are becoming increasingly common (e.g., Guyton et al. 2020; Gordon et al. 2023), understanding how these effects develop across spatial extents and temporal scales remains limited (Bakker and Svenning 2018). Addressing this knowledge gap is essential for informing the design and management of both large‐ and small‐scale rewilding initiatives, particularly given the substantial economic investment involved and the importance of maintaining public and stakeholder support.

African savannah elephants ( *Loxodonta africana* ) are mega‐herbivores and ecosystem engineers that strongly influence vegetation structure and ecosystem processes through herbivory, tree toppling, and branch breakage (Guldemond and van Aarde 2010; Guldemond et al. 2017). Historically widespread across Africa, elephants have been extirpated from large portions of their former range (Lister 2013; Chase et al. 2016). The loss of elephants has been linked to shifts in ecosystem states, including increased woody vegetation cover and homogenization of the open–closed savannah matrix (Venter et al. 2018). Conversely, elephant reintroductions can reverse these changes by increasing woody vegetation consumption and damage, highlighting their potential to restore fundamental ecological processes through trophic rewilding.

Beyond their direct impacts on vegetation, elephants can indirectly affect other fauna by modifying habitat structure and influencing predation risk (Laundré et al. 2001; Valeix et al. 2011; Burkepile et al. 2013; le Roux et al. 2018; Harris et al. 2019). For example, elephant‐induced tree damage can create hollows and cracks that provide refugia for arboreal species such as lizards (Pringle 2008), while reductions in woody plant density can increase visibility for larger mammals, enhancing predator detection and avoidance (Fauteux et al. 2012; Kuijper et al. 2013; Voysey et al. 2021). The latter represents an example of the “landscape of fear” concept, which proposes that prey change their behavior in response to perceived predation risk, with cascading effects on other ecological processes (Laundré et al. 2010). Prominent examples of these dynamics have been observed following wolf reintroductions in Yellowstone National Park (USA; see Ripple et al. (2025) for recent work).

Smaller mammalian herbivores, such as rodents, rely on dense vegetation and ground‐level structure to mitigate predation risk. Their small size allows concealment from visual predators and access to narrow refuges, and they preferentially forage and nest in areas with high vegetation or woody debris cover (Kotler et al. 1991; Morris and Davidson 2000; Brown and Kotler 2004). Coarse woody debris (CWD)—fallen logs, branches, and tree trunks—is an important component of ground‐lying habitat, providing critical refugia, nesting sites, and movement corridors for small mammals (Carey and Johnson 1995; Fauteux et al. 2012; Sullivan and Sullivan 2019). Experimental addition of ground wood has been shown to moderate small mammal abundance and activity at a community level (Sullivan et al. 2012), emphasizing its structural importance for habitat use and predator avoidance in certain situations and scales.

Rodents perform key ecosystem functions as seed predators, scatter‐hoarders, and seed dispersers, influencing seed survival and plant regeneration (Vander Wall 2002; Xiao et al. 2005; Dennis 2007; Lacher et al. 2019). Their foraging behavior affects vegetation dynamics after disturbance and contributes to broader ecosystem processes. For example, seed predation in Indonesia's deforested peatlands contributes to reducing regeneration capacity (Blackham and Corlett 2015), while rodent seed caching in Californian chaparral shrublands increases seed survival during wildfire by placing seeds below the “heat pulse kill zone”, thereby facilitating fire‐vegetation mutualisms (Peterson and Parker 2016). Rodents also serve as prey for a range of mesopredators and raptors, forming an important trophic link in terrestrial food webs (Dickman 1999; Hanski et al. 2001). Understanding how small mammals respond to environmental modifications, including those caused by elephants, is therefore critical for predicting cascading effects on ecosystem structure and function.

Here, we investigate whether elephant reintroduction (as a case of de facto trophic rewilding) indirectly influences the site‐use and behavior of a common rodent, the Natal multimammate mouse ( *Mastomys natalensis* ), through the creation of coarse woody debris. In doing so, we assess the utility of trophic rewilding as a tool for biodiversity conservation.

Elephants are major agents of coarse woody debris formation, particularly larger‐sized items, through tree felling and branch breakage (Asner and Levick 2012; Cook et al. 2017; Landman et al. 2019). The abundance of large CWD items is higher where elephants are present and have been restored and can decline with time since elephant reintroduction (Landman et al. 2019; Gordon et al. 2023). There is growing evidence that CWD influences the behavior and predation risk of larger mammals in African savannahs. For example, CWD provides habitat refugia for species such as warthogs ( *Phacochoerus africanus* ) but acts as a “predation trap” for species such as impala ( *Aepyceros melampus* ) by reducing visibility and escape efficiency (Fležar et al. 2019). However, few studies have examined this relationship for smaller species such as rodents, and fewer (if any) have explored cascading relationships between megafauna reintroduction, habitat modification, and rodent activity or behavior.

Using field surveys and a manipulative experiment, we test the hypothesis that elephant reintroduction and elephant‐induced habitat modifications provide refugia that affect rodent behavior. Specifically, we predict that:

- Coarse woody debris abundance (felled trees and large branches) will be higher at sites with elephant reintroductions than at sites without.
- Rodent site visitation and the frequency of feeding and “relaxed” behaviors will be higher within coarse woody debris patches than in adjacent open areas where elephants are present.
- Rodent “giving‐up density”—a metric quantifying landscapes of fear (Laundré et al. 2010)—will be lower in coarse woody debris patches than in open areas, reflecting greater energy allocation to feeding rather than vigilance.

## 2 Materials and Methods

### 2.1 Study Area

**FIGURE 1:**
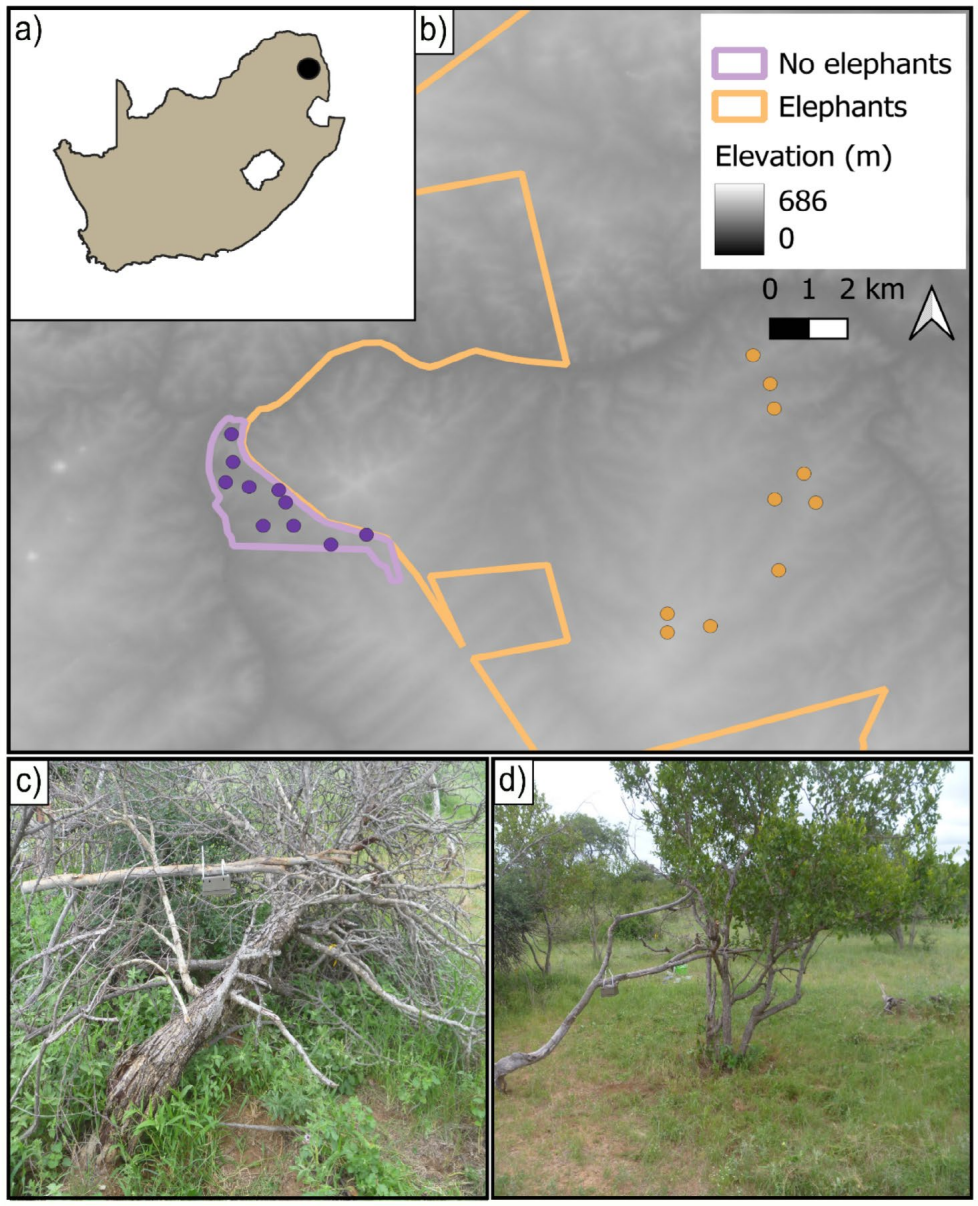
(a) Map of the study area in north‐east South Africa. (b) Location of field sites where coarse woody debris abundance was assessed between nature reserves with (orange points) and without (purple points) elephant reintroductions. Rodent activity and behavior were also assessed at eight of the sites with elephant reintroductions. (c, d) Examples of experimental treatments used to compare rodent activity and giving‐up density between coarse woody debris (c) and open (d) patches.

The study was conducted in granite lowveld savannah (Mucina et al. 2018) within Balule Nature Reserve (BNR), northeastern South Africa (Figure 1). Mean annual precipitation is 495 mm, with most rainfall occurring between October and March (the wet season) when maximum temperatures often exceed 35°C (Platts et al. 2015). The woody vegetation is dominated by marula ( *Sclerocarya birrea* ), knobthorn (*Senegalia nigrescens*), bushwillow (*Combretum* spp.), corkwood (*Commiphora* spp.), and raisin‐bush (*Grewia* spp.), with a continuous grassy understory across the region. Fire is rare due to low grass phytomass and fire exclusion (Peel 2014). Table S1 provides an overview of the biophysical and climatic conditions within the study area.

Balule Nature Reserve was established in 1993 and is part of the Association of Private Game Reserves network (2011 km^2^), which allows fauna free access across all included reserves and with the adjoining Kruger National Park (19,485 km^2^). Elephants were restored to the study area in 2003, with average elephant densities of 1.24 individuals km^−2^ recorded between 2014 and 2018 (based on dry‐season aerial census counts). A suite of up to 148 native herbivores and carnivores occur across the reserves (SanParks 2026), including the largest herbivores and carnivores such as African wild dog ( *Lycaon pictus* ), black and white rhinoceros ( *Ceratotherium simum* , *Diceros bicornis* ), cape buffalo ( *Syncerus caffer* ), elephant, giraffe ( *Giraffa camelopardalis* ), leopard ( *Panthera pardus* ) and lion ( *Panthera leo* ). We treat elephant reintroductions in BNR as de facto trophic rewilding because elephants' strong ecological effects occur largely in the absence of human intervention and are self‐sustaining (Robson and van Aarde 2018).

Two fully fenced properties adjoining Balule Nature Reserve were granted conservation status around 1993 and were subsequently incorporated into the reserve estate. A similarly diverse assemblage of herbivores and carnivores occurs within these reserves compared with BNR (SanParks 2026); however, white rhinoceros, black rhinoceros, cape buffalo, elephant, and lion are absent. Reserve managers suggest that leopard and African wild dog occur at densities comparable to those in BNR. We treated these fenced reserves as experimental “control” areas where elephant reintroductions have not occurred. Although several larger species were absent from these reserves, elephants are the only species known to fell trees within the study region (Asner and Levick 2012; Cook et al. 2017; Landman et al. 2019). Therefore, we are confident that elephant presence or absence was the primary factor contributing to differences in coarse woody debris.

### 2.2 Elephant Rewilding and Coarse Woody Debris Density

The density of large coarse woody debris items, representing fallen or felled trees and large branches, was assessed at 10 sites within reserves with and without elephant reintroductions (20 sites total), following the approach of Gordon et al. (2023; Figure 1). Sites were located on catena hilltops 100–200 m from small access roads and spaced 1–4 km apart at both reserves. All medium‐ (> 5 cm and < 20 cm diameter at intercept) and large‐sized (≥ 20 cm diameter at intercept and ≥ 200 cm total length) coarse woody debris items were recorded at 1 m intervals along three 50 m transects positioned at the ends and middle of a 50 m × 20 m quadrat using a 1 m pole. The total number of medium‐ and large‐sized coarse woody debris intercepts per site was used as the replicate unit in analyses. Coarse woody debris is not collected by humans at any of the study sites.

Bayesian generalized linear models were used to determine how medium‐sized (Gaussian distribution; raw CWD data approximated a normal distribution) and large‐sized (negative binomial distribution; raw CWD data was right‐skewed, with overdispersion observed in a preliminary Poisson model) coarse woody debris abundance varied between reserves with and without elephants using the “stan_glm” function in the “rstanarm” package in R (Goodrich et al. 2020).

Models were fitted with four chains of 2000 iterations (1000‐iteration burn‐in) and uninformative priors. Model diagnostics (e.g., chain mixing, autocorrelation, and effective sample size) were assessed using the “launch_shinystan” function. Model convergence and fit were further evaluated using posterior predictive checks, visual inspection of trace plots, effective sample sizes, Gelman–Rubin convergence diagnostics (R‐hat), and Hamiltonian Monte Carlo sampler diagnostics (Gelman et al. 2020; Vehtari et al. 2021). All models showed adequate convergence (all R‐hat values < 1.01), large effective sample sizes, good chain mixing, and no divergent transitions (Table S2). Posterior predictive checks indicated that simulated data adequately reproduced observed data distributions.

Model inference was based on zero‐centered credible intervals, with evidence for a robust defensible effect inferred when the central 90% credible interval did not overlap zero. We used 90% credible intervals because Bayesian credible intervals are not inherently linked to the conventional 95% threshold used in frequentist null hypothesis testing. In Bayesian inference, interval widths may be selected according to inferential goals, and 90% credible intervals are commonly used in Bayesian workflows, including as the default interval width in the “rstanarm” package used for model fitting. Exclusion of zero from a central 90% credible interval indicates that > 95% of the posterior probability support either a positive or negative effect, providing evidence for a strong, robust and consistent directional effect. For simplicity, we define any responses where the 90% credible intervals do not cross zero as a “significant” effect.

### 2.3 Rodent Site Visitation and Behavior

Rodent site visitation and behavior were assessed using trail cameras (Scoutguard Boly SG2060‐K) placed within a fallen tree (hereafter “coarse woody debris”) and in an adjacent open area 10 m from the outer edge of the tree (hereafter “open”) at eight sites in Balule Nature Reserve where elephants were present (Figure 1). Visual assessment suggested that all trees were felled by elephants, evidenced by lower bark damage on the trunk. Sites were spaced at approximately 1 km intervals.

Trail cameras were mounted 1 m aboveground facing downward and set to record 10 s video clips with a 1 min delay between clips during the night (dusk to dawn) when rodents are active. Twenty peanuts and a bait (a 2 cm muslin ball with peanut butter scent) were placed at the centre of the camera's focal view to attract rodents. Activity was monitored over a 16‐day period (21 January–6 February 2020), with baits replaced every 3 days. Rodent site visitation was quantified as the total number of video clips in which rodents were observed per treatment per site. To ensure independence, only observations > 1 h apart were included. Visual inspection of camera images confirmed that > 90% of rodents were *Mastomys natalensis* , and we focus on these observations only.

Feeding and “relaxed” behaviors were assessed using clips from the final 4 days of sampling, when rodent activity was highest. Feeding was defined as periods when individuals were consuming peanuts or interacting with the bait. Resting was defined as when individuals were stationary with both hind legs on the ground for > 3 s or engaged in grooming. We attempted to assess vigilance—defined as abrupt deviations from normal activity, typically a freezing posture with an attentive gaze—but this behavior was observed at only three sites and was excluded from analysis. Feeding and resting events were quantified as the number of clips showing each behavior per treatment per site during the four‐day period.

The density of all woody plants ≥ 1 m tall and coarse woody debris items ≥ 2 m long and ≥ 50 cm high was recorded within a 15 m radius of each camera.

Bayesian generalized linear mixed‐effects models with Poisson distribution were used to assess how site visitation and feeding and resting behaviors varied between coarse woody debris and open treatments using the “stan_glmer” function in rstanarm. Woody vegetation and coarse woody debris density were included as fixed effects, and site was included as a categorical random factor. Models were fit using the protocol described in Section 2.2, with all models showed adequate model fit diagnostics (Table S2).

### 2.4 Rodent Giving‐Up Density

Giving‐up density (GUD) represents the amount of food remaining in a foraging patch when an animal ceases feeding, reflecting the balance between foraging benefits and associated costs from predation risk or energetic expenditure (Laundré et al. 2010). Lower GUD values indicate more efficient foraging or lower perceived risk, while higher GUD values suggest reduced foraging efficiency or heightened perceived risk.

Giving‐up density trials were conducted for three consecutive nights alongside the camera surveys (19–23 January 2020) by placing three sand‐filled feeding trays, spaced 5 m apart, within each open and coarse woody debris treatment per site. Thirty peanut halves were placed in each tray in the mid‐afternoon and mixed into the sand. The number of peanuts remaining the next morning was counted, and trays were replenished to 30 each day. Giving‐up density was calculated as the total number of seeds remaining across the three trays per day per treatment and was used as the replicate unit for analysis.

Bayesian generalized linear mixed‐effects models with a Gaussian distribution were used to test how GUD varied between coarse woody debris and open treatments, with sample date nested within site as a random factor. Models were fit using the protocol described in Section 2.2, with all models showed adequate model fit diagnostics (Table S2).

## 3 Results

**FIGURE 2:**
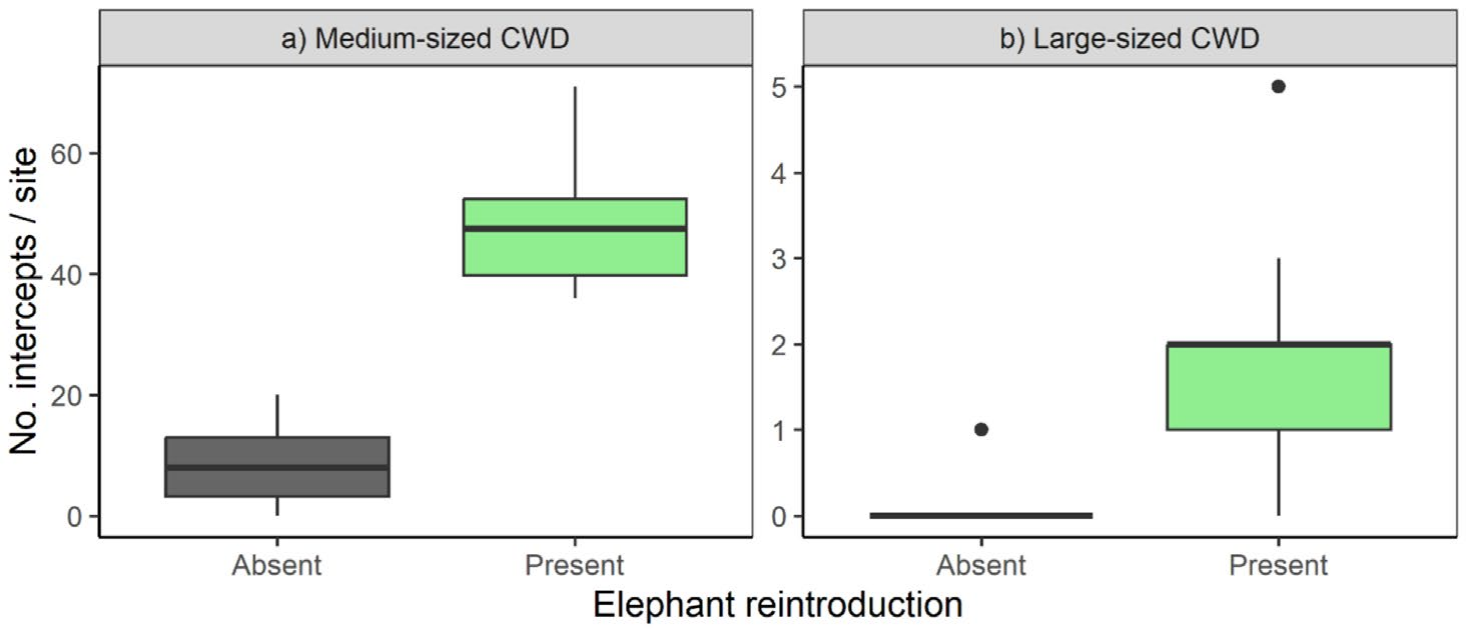
The number of (a) medium‐ and (b) large‐sized coarse woody debris intercepts recorded along 50 m transects in conservation areas where elephants were absent versus present (restored). Boxplots show median values (horizontal line), the extent of the central 50th (box) and 95th (vertical lines) percentiles and outlying data points (points).

The abundance of medium‐ and large‐sized coarse woody debris items was significantly higher in areas where elephants were present than absent, with mean differences of 39.8 (elephant absent = mean 8.71 ± SD 6.92, elephant present = 48.55 ± SD 10.94) and 1.7 (elephant absent = mean 0.14 ± SD 0.31, elephant present = 1.82 ± SD 1.47) intercepts per site, respectively (Table 1, Figure 2).

**TABLE 1:** Mean coefficient estimates (±central 90th percentile credible intervals) from Bayesian models determining how (a) medium‐ and large‐sized coarse woody debris abundance varied between conservation reserves where elephants were absent and restored, and (b) rodent site visitation, feeding event frequency, resting event frequency and giving up density varied between coarse woody debris and opened experimental treatments.

Dependent variable	Independent variable		
**(a) Coarse woody debris abundance vs. elephant presence**			
	**Elephant restoration**		
Medium‐sized CWD	39.7 ± 32.4 to 46.9^a^	—	—
Large‐sized CWD	3.1 ± 1.5 to 5.0^a^	—	—
**(b) Rodent behavior vs coarse woody debris treatment**			
	**Treatment**	**Woody vegetation**	**Coarse woody debris**
Rodent site‐visitation	−0.5 ± −0.8 to −0.3^a^	0.0 ± −0.1 to 0.1	0.0 ± −0.5 to 0.5
Rodent feeding events	−0.6 ± −1.1 to −0.1^a^	0.0 ± −0.1 to 0.2	0.3 ± −0.3 to 1.0
Rodent resting events	−2.1 ± −3.2 to −1.1^a^	0.1 ± −0.1 to 0.2	0.4 ± −0.1 to 1.0
Giving up density	6.2 ± 0.6 to 11.7^a^	−0.4 ± −1.0 to 0.3	−5.3 ± −9.1 to −1.5^a^

*Note:* —: Shows cells where an independent variable was not present.

^a^ A “significant” effect was detected if the central 90% credible intervals did not cross zero.

**FIGURE 3:**
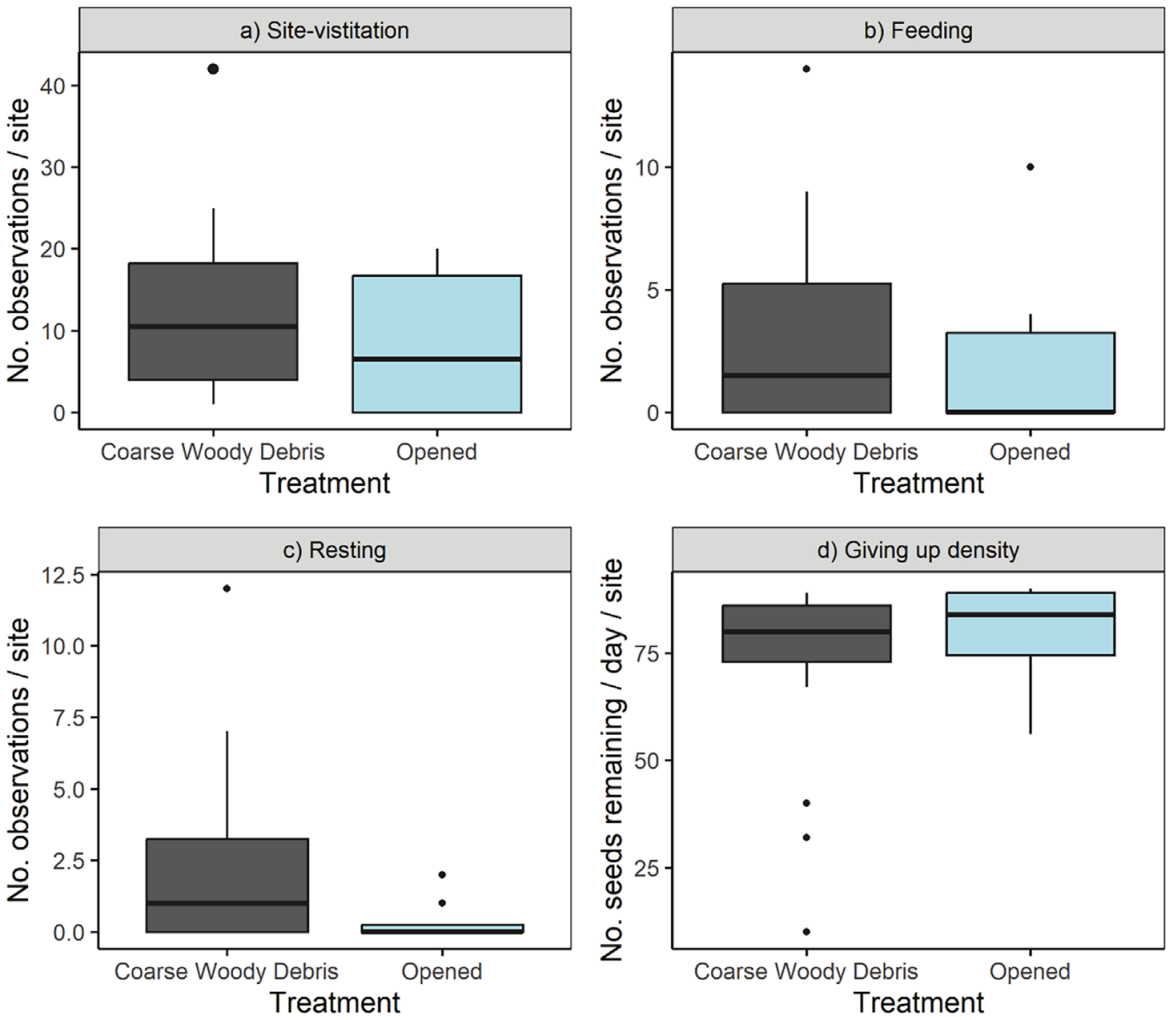
(a) Rodent site‐visitation, (b) feeding behavior frequency, (c) resting behavior frequency and (d) giving up density between experimental treatments located in coarse woody debris and opened patches. Boxplots show median values (horizontal line), the extent of the central 50th (box) and 95th (vertical lines) percentiles and outlying data points (points).

Rodent site visitation and the frequency of feeding and resting events were significantly higher within the coarse woody debris than the open treatment, with mean differences of 5.6 rodent observations (CWD treatment = mean 14.12 ± SD 13.70, opened treatment = 8.50 ± SD 8.97), 1.6 feeding events (CWD treatment = mean 3.75 ± SD 5.14, opened treatment = 2.12 ± SD 3.56), and 2.5 resting events per site (CWD treatment = mean 2.87 ± SD 4.35, opened treatment = 0.37 ± SD 0.74; Table 1, Figure 3a–c). Neither woody vegetation nor coarse woody debris density independently affected visitation or behavior frequencies (Table 1).

Rodent giving‐up density was significantly higher in the open than in the coarse woody debris treatment, with a mean difference of six seeds per site per tray (CWD treatment = mean 74 ± SD 19, opened treatment = 80 ± SD 11; Table 1, Figure 3d). Beyond the treatment effect, coarse woody debris density showed a significant negative association with giving‐up density (Table 1).

## 4 Discussion

### 4.1 Elephant Rewilding, Coarse Woody Debris, and Rodent Behavior

Our findings support the hypothesis that elephant rewilding indirectly affected the behavior of a common rodent by altering habitat structure. Coarse woody debris density was significantly higher at sites where elephants were present, consistent with prior studies identifying elephants as key agents of wood production through tree felling and branch breakage (Asner and Levick 2012; Cook et al. 2017; Landman et al. 2019; Gordon et al. 2023). In turn, these structural legacies translated to altered rodent habitat use: the Natal multimammate mouse exhibited higher site visitation, feeding, and resting within CWD patches compared with adjacent open areas. Together, these results support the hypothesis that elephants act as ecosystem engineers (Jones et al. 1994; Guldemond and van Aarde 2010) whose influence cascades to fauna at lower trophic levels.

The increased availability of CWD associated with elephant presence likely provided both cover from predators and greater microhabitat availability, enabling rodents to forage and rest with reduced predation risk (Morris and Davidson 2000; Brown and Kotler 2004). Ground wood also provides important nesting sites and food resources, including seed caches, which are used by many African savannah rodents to persist during dry season conditions (Kuechly et al. 2011). Similar behavioral shifts have been observed in North American and European systems where large herbivores restructure understorey conditions and influence rodent assemblages (Smit et al. 2001; Munoz et al. 2009), and in Australian deserts, where understory structure comparable to CWD affects rodent activity and vigilance (Gordon et al. 2015). Collectively, these studies—and ours—support the general role of CWD and ground wood as habitat refugia for rodents.

The reduced giving‐up densities observed within coarse woody debris patches indicate that rodents perceived these microhabitats as safer and were willing to invest more foraging effort before abandoning a food patch. This aligns with the “landscape of fear” framework (Laundré et al. 2010), which posits that animals balance foraging and vigilance according to perceived predation risk. By reducing open‐ground exposure, CWD likely lowers visual detection by predators such as owls, mongooses, and snakes, allowing rodents to reallocate energy toward feeding and resting. These behavioral adjustments parallel patterns seen in temperate forests, where coarse woody debris enhances small mammal foraging efficiency and local abundance (Carey and Johnson 1995). Our findings extend this concept to African savannahs, showing that structural refugia generated by megaherbivores can reshape fine‐scale landscapes of fear.

The indirect effects of elephant activity on rodents may have broader implications for ecosystem functioning through cascading pathways. By promoting rodent foraging within CWD‐rich areas, elephants may influence spatial patterns of seed predation and dispersal, potentially affecting plant regeneration trajectories following disturbance (Vander Wall 2002; Xiao et al. 2005; Dennis 2007). If elephants increase the heterogeneity of microhabitats where rodents feed and cache seeds, they could enhance vegetation mosaic complexity, reinforcing the open–closed structural diversity characteristic of intact savannahs (Kuechly et al. 2011; Skowno et al. 2017). Conversely, in the absence of elephants, reduced woody debris may heighten predation risk for rodents, suppressing their activity and limiting their role as seed dispersers, further amplifying vegetation homogenization. Rodents also provide key prey resources for other fauna (Dickman 1999; Hanski et al. 2001) and therefore indirectly influence predator populations. Such cascading feedbacks underscore how rewilding megaherbivores can restore not only structural but also functional components of savannah ecosystems.

While our study provides strong evidence that elephant‐induced habitat modifications affect rodent behavior, several factors could have influenced our results. First, we only assessed rodent site visitation and behavior within the reserve where elephants were present. However, our results should also be applicable to the reserve where elephants were absent because both toppled trees and carnivores known to consume rodents occurred across all reserves, meaning that the key factors constraining site visitation and behavior were present across the study area. Second, we used baited camera traps to increase site visitation, which could have influenced rodent behavior. However, this is common practice in ecological monitoring programs (Hopkins et al. 2024), and preliminary trials using unbaited cameras failed to detect any rodents. Therefore, baited cameras were necessary to allow meaningful inference. Third, processes other than elephant damage could have contributed to tree toppling. To account for such effects, we positioned study sites close to one another within comparable climatic and biophysical landscapes. Consequently, although other processes such as local weather and climate may influence coarse woody debris (CWD) formation, these factors were controlled for through the study design. Furthermore, elephant damage is the only biological process (at least for vertebrates; e.g., see Davies et al. (2016) for important role of termites) known to fell trees within the study area (Asner and Levick 2012; Cook et al. 2017; Landman et al. 2019). Therefore, we argue that our study design adequately accounted for this potential source of confounding.

Although we do not believe that the above factors greatly influenced inference in our study, future research explicitly addressing these issues would further strengthen our conclusions. We also suggest that future research could address several questions that our study was not able to resolve. For example, the extent to which the behavioral responses observed here translate into demographic or population‐level outcomes, whether predator communities adapt to these altered landscapes, or if elephants generate spatially heterogeneous effects depending on vegetation type, fire history, and time since reintroduction (Davies et al. 2018; Landman et al. 2019; Gordon et al. 2023).

### 4.2 Management Implications

Our results suggest that elephant rewilding initiates a cascade of ecological effects extending from vegetation structure to small mammal behavior. By generating coarse woody debris, elephants create spatial refugia that reduce predation risk and modify foraging landscapes for rodents. These behavioral shifts have the potential to reverberate through food webs, influencing both plant regeneration and predator–prey interactions. Our findings highlight how the restoration of megafaunal processes can reinstate lost ecological functions and emphasize the importance of considering behaviourally mediated pathways when evaluating trophic rewilding outcomes in savannah ecosystems.

Elephant reintroductions—and trophic rewilding more broadly—should be viewed not only as efforts to restore population viability but also as mechanisms to reinstate trophic processes that regulate ecosystem structure and function (Svenning et al. 2016, 2019). By enhancing woody debris formation and associated refugia, elephants contribute to multi‐trophic resilience, linking vegetation heterogeneity, small‐mammal behavior, and predator dynamics. Demonstrating this, our results suggest that to truly quantify “success,” rewilding programs require long‐term monitoring of multiple ecological responses across trophic levels. For example, coarse woody debris densities, small mammal activity, and seedling recruitment. Developing such integrative frameworks will be crucial for managing and evaluating future megafaunal rewilding initiatives.

## Author Contributions

**Christopher E. Gordon:** conceptualization (equal), data curation (lead), investigation (lead), methodology (equal), validation (lead), visualization (lead), writing – original draft (lead), writing – review and editing (equal). **Paul Allin:** data curation (supporting), investigation (supporting), methodology (supporting), writing – review and editing (supporting). **Michelle Greve:** conceptualization (equal), methodology (supporting), project administration (supporting), writing – original draft (supporting), writing – review and editing (equal). **Jens‐Christian Svenning:** conceptualization (equal), funding acquisition (lead), methodology (equal), resources (lead), supervision (lead), writing – original draft (supporting), writing – review and editing (equal).

## Funding

This work was supported by the South African National Research Foundation (116333), Danmarks Grundforskningsfond (DNRF173), Carlsberg Foundation Semper Ardens (CF16‐0005), Villum Fonden (16549), and Research Fund Denmark (0135‐00225B).

## Conflicts of Interest

The authors declare no conflicts of interest.

## Supporting information


**Table S1:** Mean values (±central 95th percentiles) for bioclimatic variables thought to impact flora and fauna within sections of Balule nature reserve where elephants were absent and present. All sites were located in Granite Lowveld savannah (Mucina et al. 2018). Data derived from Gordon et al. (2023).
**Table S2:** Diagnostic values from generalized linear and generalized linear mixed‐effects models assessing how various predictor variables influence: (a) medium‐sized coarse woody debris (CWD) abundance, (b) large‐sized CWD abundance, (c) rodent site visitation, (d) rodent feeding event frequency, (e) rodent resting event frequency, and (f) rodent giving‐up density. R‐hat is the Gelman–Rubin convergence statistic assessing model convergence, with values < 1.01 indicating adequate chain mixing. Bulk and tail Effective Sample Size (ESS) values assess whether there are sufficient effective samples to adequately represent processes within the central portion (bulk) and extreme ends (tail) of the posterior distribution, with values > 400 generally considered acceptable. The number of divergent transitions assesses whether the Hamiltonian Monte Carlo (HMC) sampler implemented in the rstanarm package was able to accurately explore the posterior distribution geometry, with 0 divergent transitions indicating well‐fit models.

## Data Availability

All the required data and script are uploaded as Supporting Information.
