# Supplementary material for: Elephant Rewilding Indirectly Affects Rodent Site‐Use and Behavior by Moderating Coarse Woody Debris Density
Source: Ecol Evol. 2026 Aug 3;16(8):e73928. doi: 10.1002/ece3.73928 (PMC13433147; doi:10.1002/ece3.73928)
Supplement: Supplementary file 1 [file ECE3-16-e73928-s001.docx]

**Elephant rewilding indirectly affects rodent site-use and behavior by moderating coarse woody debris density**

*Christopher E Gordon^1,2^, Paul Allin^3^, Michelle Greve^4^ and Jens-Christian Svenning^2^*

1. Hawkesbury Institute for the Environment, Western Sydney University, Locked Bag 1797, Penrith, NSW, 2751, Australia

2. Center for Ecological Dynamics in a Novel Biosphere (ECONOVO), Department of Biology, Aarhus University, 8000 Aarhus C, Denmark

3. Transfrontier Africa, Hoedspruit, South Africa, 1380

4. Department of Plant and Soil Sciences, University of Pretoria, Pretoria, South Africa, 0028

*Corresponding author:* Christopher E. Gordon, email- chris.gordon@westernsydney.edu.au

**Supporting information**

**Table S1.** Mean values (± central 95^th^ percentiles) for bioclimatic variables thought to impact flora and fauna within sections of Balule nature reserve where elephants were absent and present. All sites were located in Granite Lowveld savannah (Mucina et al. 2018). Data derived from Gordon et al. (2023).

| **Reserve** | **Size (km^2^)** | **Elephants introduced**  **(year)** | **Access: Kruger NP** | **Elephant density**  **(ind/km^2^)** | **MAP**  **(mm)** | **MPWM**  **(mm)** | **Elevation**  **(m)** | **Distance to water**  **(m)** | **No. fires:**  **2000–2016**  **(count)** |
| --- | --- | --- | --- | --- | --- | --- | --- | --- | --- |
| Elephant absent | 559 | None | No | 0 | 507  (507–507) | 87  (87–87) | 429  (436–512) | 1.5  (1.7–4.2) | 0 |
| Elephant present | 7.5 | 2003 | Yes | 1.24  (0.7–1.62) | 456  (445–465) | 85  (84–86) | 466  (436–512) | 3.0  (1.7–4.2) | 0 |

Aerial census counts between 2014 – 2018 were used to assess dry season elephant density. ARFICLIM (~9 km resolution; Platts et al. 2015) was used to assess mean annual precipitation (MAP) and mean precipitation in the wettest month (MPWM). Shuttle Radar Topography Mission data was used to assess elevation using the USGS’s Earth Explorer interface (30 m resolution). The Global Surface Water database’s occurrence layer (~25 m resolution; Pekel et al. 2016) was used to assess distance to the closest persistent water source (Distance to water). Persistent water was defined as areas where water was observed on > 20 % of monthly satellite images between 1984 – 2016. Data layers derived from MODIS satellite imagery (Giglio et al. 2015) were used to assess fire frequency (~450 m resolution) between 2000 and 2016.

**Table S2.** Diagnostic values from generalized linear and generalized linear mixed-effects models assessing how various predictor variables influence: a) medium-sized coarse woody debris (CWD) abundance, b) large-sized CWD abundance, c) rodent site visitation, d) rodent feeding event frequency, e) rodent resting event frequency, and f) rodent giving-up density. R-hat is the Gelman–Rubin convergence statistic assessing model convergence, with values < 1.01 indicating adequate chain mixing. Bulk and tail Effective Sample Size (ESS) values assess whether there are sufficient effective samples to adequately represent processes within the central portion (bulk) and extreme ends (tail) of the posterior distribution, with values > 400 generally considered acceptable. The number of divergent transitions assesses whether the Hamiltonian Monte Carlo (HMC) sampler implemented in the rstanarm package was able to accurately explore the posterior distribution geometry, with 0 divergent transitions indicating well-fit models.

| **Predictor variable** | **R-hat** | **ESS bulk** | **ESS tail** | **No. divergent trends** |
| --- | --- | --- | --- | --- |
| a) Medium-sized coarse woody debris | | | | |
| Elephant presence | 1 | 2961 | 2242 | 0 |
| b) Large-sized coarse woody debris | | | | |
| Elephant presence | 1 | 1490 | 1046 | 0 |
| c) Rodent site visitation | | | | |
| CWD treatment | 1 | 4468 | 2767 | 0 |
| Shrub density | 1 | 1928 | 1967 | 0 |
| CWD density | 1 | 1807 | 1753 | 0 |
| d) Rodent feeding event | | | | |
| CWD treatment | 1 | 5363 | 2999 | 0 |
| Shrub density | 1 | 1614 | 2030 | 0 |
| CWD density | 1 | 1768 | 1501 | 0 |
| e) Rodent resting events | | | | |
| CWD treatment | 1 | 3551 | 2359 | 0 |
| Shrub density | 1 | 1596 | 1884 | 0 |
| CWD density | 1 | 1554 | 1729 | 0 |
| f) Rodent giving up density | | | | |
| CWD treatment | 1 | 7991 | 2794 | 0 |
| Shrub density | 1 | 2837 | 2409 | 0 |
| CWD density | 1 | 3073 | 2345 | 0 |
